# Determination of Naproxen Impurities’ Relative Response Factors by qNMR-HPLC Using DMSO-*d*_6_ Solvent as an Internal Calibrant

**DOI:** 10.3390/molecules31203598

**Published:** 2026-10-10

**Authors:** Hengting Pu, Yang Liu

**Affiliations:** 1 National Institutes for Drug Control, Beijing 102629, China; ppu0907@163.com; 2 School of Pharmaceutical Sciences, Yantai University, Yantai 264005, China

**Keywords:** qNMR, HPLC, relative response factors, naproxen, drug impurities, internal calibrant

## Abstract

Quantitative nuclear magnetic resonance coupled with HPLC (qNMR-HPLC) is a rapid and non-destructive approach. It does not require accurate sample weights and purification of reference substances. However, when determining the relative response factors (RRFs) of multiple drug impurities, spectral overlap may be a problem. As naproxen and its impurities are similar in structure, it is difficult to determine the corresponding characteristic signal peaks of each component in the ^1^H NMR spectrum of the principal component and impurities. This study aimed to develop a qNMR-HPLC method using the δ 2.5 ppm signal of DMSO-*d*_6_ as an internal calibrant to determine the RRFs of six naproxen impurities (I–VI). Each component was analyzed by qNMR separately and the ratio relationship was linked by DMSO-*d*_6_ calibrant. The obtained RRFs (0.30, 0.76, 1.14, 1.00, 0.59, and 0.51, respectively) were consistent with those determined using the HPLC standard curve method (0.30, 0.78, 1.04, 1.05, 0.59, and 0.51, respectively). The method showed a linear R^2^ of 1 in the 1–12 mg·mL^−1^ range, the RSD precision of five replicates was 0.10%, and the RSD of solution stability within 24 h was 0.10%. The method is well suited for scenarios involving minimal sample quantities that cannot be accurately weighed and for the simultaneous determination of multiple impurity RRFs in cases where spectral overlap is a challenge.

## 1. Introduction

Impurities in drugs are generated during drug production, transportation, and storage. They can affect the efficacy and safety of drugs. The strict control of impurity levels is necessary to ensure drug safety and avoid potential side effects. Impurities are categorized as organic, inorganic, and residual solvents by the International Council for Harmonization of Technical Requirements for Pharmaceuticals for Human Use (ICH) [1]. Organic impurities mainly include starting materials, byproducts, intermediates, and degradation products. The inorganic impurities in the production process mainly include reagents, catalysts, heavy metals, and other residual metals [1]. Normally, organic impurities are more complicated to separate than inorganic impurities or residual solvents.

The structures of organic impurities are typically determined using mass spectrometry (MS), nuclear magnetic resonance (NMR), and infrared (IR) and ultraviolet (UV) spectroscopy [2,3,4,5,6]. A common method for determining organic impurity content is HPLC, which includes the area normalization, internal standard, external standard, and active pharmaceutical ingredient (API) self-referencing (with or without a correction factor) methods [7]. Among them, the external standard method does not need to consider the relative response factors (RRFs) of impurities and can be used to analyze various impurities; however, it requires impurity reference standards. The preparation of reference standards is intricate and challenging and involves raw material procurement, uniformity inspection, stability testing, assignment determination, packaging and storage, and quality monitoring, among other processes [8,9,10]. Furthermore, the impurity reference standards used for content determination typically need to guarantee a purity of no less than 95% [10,11]. Therefore, when impurity reference standards are unavailable, the drug impurity content cannot be determined using the HPLC external standard method.

Both the HPLC self-referencing method without a correction factor and the one with a correction factor do not require impurity reference standards. However, the former ignores that impurities and principal components may have different RRFs, which leads to deviations in the detection results from the actual values. The introduction of RRFs in the latter method renders the analysis of impurity content more accurate and has been widely used to determine the content of related substances [12]. This method has also been applied to national pharmacopeias. Both the Chinese and European Pharmacopoeia (EP11.0) define it as a correction factor, whereas the United States Pharmacopoeia defines it as an RRF, which is the inverse of the relationship between the two. When the correction factor exceeds 0.9–1.1 [13] (or the range of 0.8–1.2 as per the European Pharmacopoeia) [14] and the self-referencing method is used, it is necessary to adopt the method with an RRF to correct the absorbance difference in the impurity and API under the same wavelength. Self-referencing with the RRF has been widely adopted in all major pharmacopoeia. To facilitate content calculation, the RRF values of certain impurities are listed in the pharmacopoeia.

The traditional method for calculating an RRF is the HPLC standard curve method, where the concentration of the main component or impurities is on the x-axis, and the corresponding HPLC peak area is on the y-axis for linear regression analysis [7]. The RRF is calculated using the slope ratio of the linear equation. However, because of the difficulty in preparing impurity reference standards and their high costs, there are challenges in using HPLC for RRF determination when impurity reference standards cannot be obtained, the sample mass is minimal, or the content is inaccurate.

NMR techniques have been widely used for the structural analysis of organic compounds [15,16]. With the development of high-magnetic-field NMR, the Fourier transformation, and chemometrics, quantitative NMR (qNMR) has rapidly developed and is used in chemistry, biology, and pharmaceutical analysis. qNMR-HPLC can accurately determine RRF impurities without precise weighing [17,18,19,20].

Previously described qNMR-HPLC methodologies require at least one resonance signal of analyte 1 to be fully resolved from those of analyte 2. Signal overlap commonly arises in cases where analyte 1 and analyte 2 have highly similar chemical structures. Under such circumstances, most NMR signals originating from the two analytes show close chemical shifts, making accurate quantification infeasible, which restricts the applicability of the qNMR-HPLC approach. Employment of an internal calibrant provides a potential solution. Given that the calibrant may also yield one or more chromatographic peaks in HPLC experiments, both qNMR acquisition parameters and HPLC chromatographic conditions have to be taken into consideration during method development.

DMSO-*d*_6_ is a widely used deuterated solvent in NMR experiments. The residual protonated DMSO signal at δ 2.5 ppm can function as an internal calibrant. Meanwhile, DMSO has almost no retention on most columns used in HPLC and is always eluted in void time; it does not interfere with the analytes in HPLC. Thus DMSO-*d*_6_ is an excellent candidate as both qNMR-HPLC solvent and internal calibrant.

**Figure 1:**
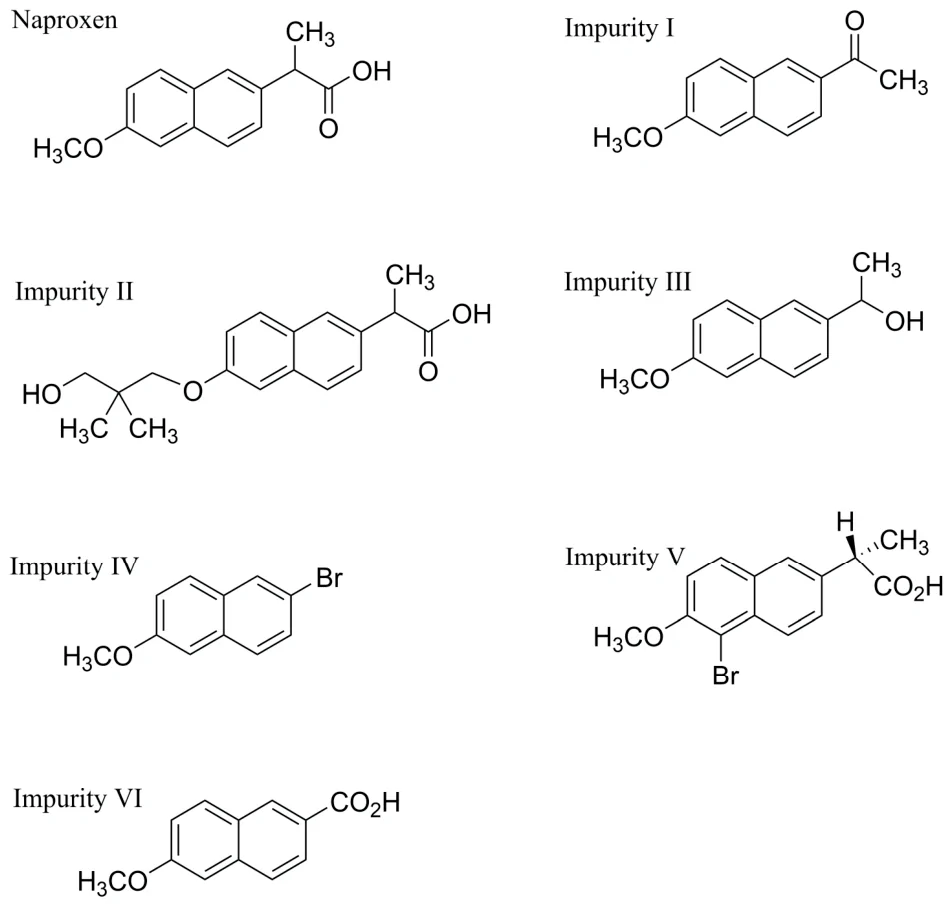
Structures of naproxen and impurity I, II, III, IV, V, and VI.

Naproxen is widely prescribed for the treatment of pain and inflammatory diseases [21,22,23]. Naproxen contains multiple impurities (I–VI; Figure 1) that can be controlled using HPLC. It is essential to measure each RRF for all six impurities. Because the structures of naproxen and its impurities are similar, multiple components in one NMR tube result in severe spectral overlap, making identifying suitable quantification signals challenging. Here, we propose and test a method based on qNMR-HPLC utilizing the ^1^H residual signal of deuterated DMSO at δ 2.5 ppm as an internal calibrant. The RRF of naproxen impurities was calculated, and methodological studies were carried out to verify the feasibility and accuracy of the method.

## 2. Results

### 2.1. Methodology of qNMR-HPLC Using ^1^H Residual Signal of Deuterated DMSO as an Internal Calibrant

Conventional HPLC determination of RRF requires knowledge of the exact concentration of naproxen and the impurities to be measured. The RRF was obtained using Equation (1) based on the accurate purity (P_n_), weight (W_n_), and peak area (A_n_) of naproxen, as well as the accurate purity (P_imp_), weight (W_imp_), and peak area (A_imp_) of the impurities. In many cases, the exact weight (W) and purity (P) were difficult to determine, making it challenging to calculate the RRF using traditional HPLC methods.

(1) $$\mathrm{RRF}\;=\frac{W_{n}\;\times {\;P}_{n}\;\times \;A_{\mathrm{imp}}}{W_{\mathrm{imp}}\;\times \;P_{\mathrm{imp}\;}\times \;A_{n}}$$

In qNMR, the relationship between the purity (P_imp_) and weight (W_imp_) of the impurities and the purity (P_n_) and weight (W_n_) of naproxen can be expressed by the peak integral (I), nuclei number (N), and relative molecular weight (M), as shown in Equation (2).

(2) $$\frac{W_{\mathrm{imp}}\;\times \;P_{\mathrm{imp}}}{W_{n}\;\times \;P_{n}}=\frac{I_{\mathrm{imp}}\;\times \;N_{n}\;\times \;M_{\mathrm{imp}}}{I_{n}\;\times {\;N}_{\mathrm{imp}}\;\times \;M_{n}}$$

Equations (1) and (2) results are combined in Equation (3) [18,19]. The RRF calculation process only requires knowledge of A, I, M, and N of naproxen and impurities.

(3) $$\mathrm{RRF}\;=\frac{I_{n}\;\times {\;N}_{\mathrm{imp}}\;\times \;M_{n}\;\times {\;A}_{\mathrm{imp}}}{I_{\mathrm{imp}}\;\times \;N_{n}\;\times \;M_{\mathrm{imp}}\;\times \;A_{n}}$$

**Figure 2:**
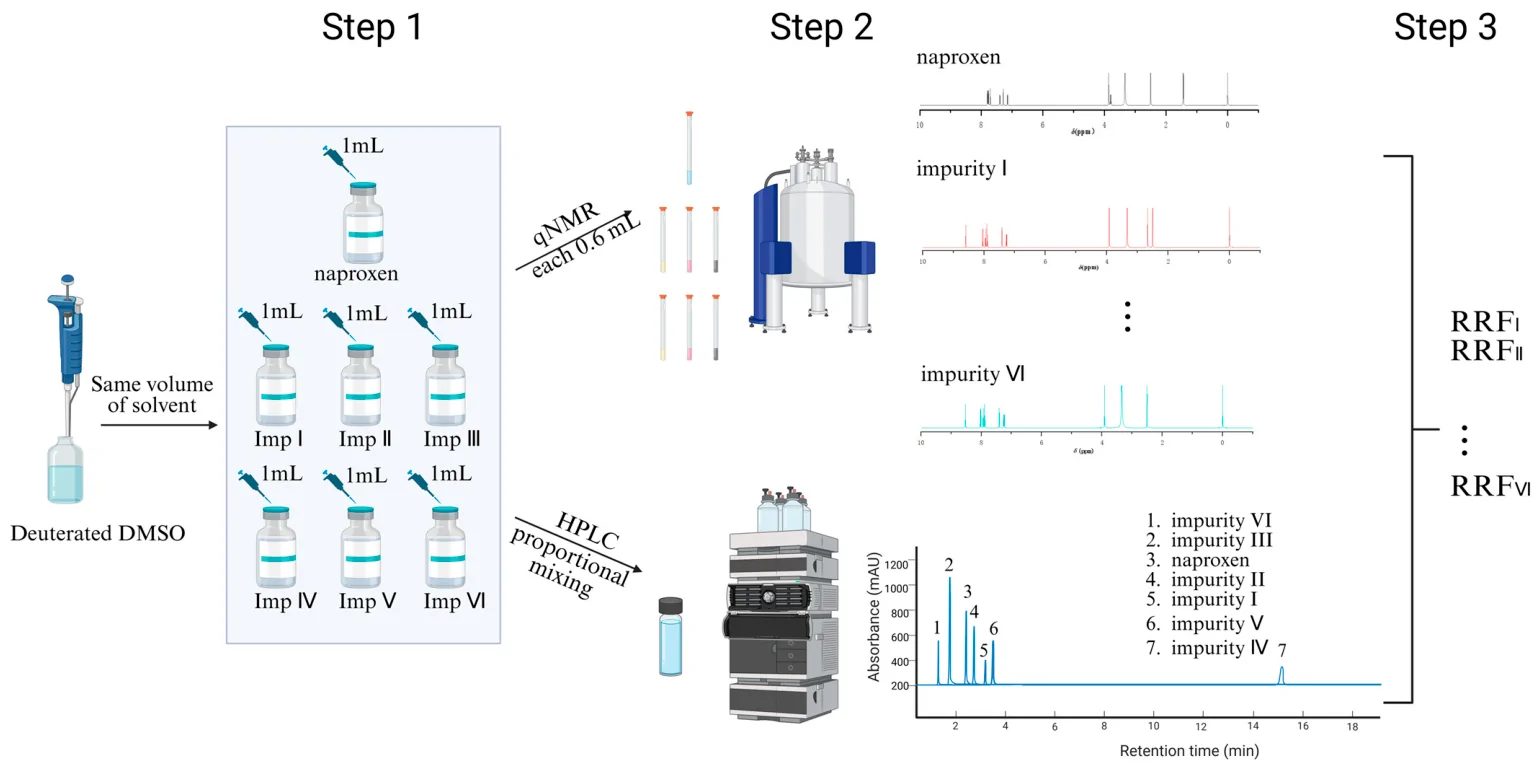
Sample preparation and calculation procedures.

However, when measuring the RRFs of multiple naproxen impurities in the same mixed solution, it is difficult to identify a suitable quantitative peak in the highly overlapping ^1^H NMR spectra, making it challenging to determine the I value using Equation (3). The principle of qNMR-HPLC using ^1^H residual signal of deuterated DMSO as an internal calibrant method is shown in Equations (4) and (5). In Step 1 (Figure 2), equal volumes of deuterated DMSO were added to the naproxen and the impurities. In the qNMR experiment, the relationship between P_(dmso,imp)_ and W_(dmso,imp)_ for both DMSO and the impurities could be converted into the ratios of I_(dmso,imp)_, N_(dmso,imp)_, and M_(dmso,imp)_, as shown in Equation (4). The relationship between P_(dmso,n)_ and W_(dmso,n)_ for both DMSO and naproxen can be converted into ratios I_(dmso,n)_, N_(dmso,imp)_, and M_(dmso,n)_, as shown in Equation (5). During the calculation, since the amount of DMSO-*d*_6_ used were the same in different NMR vials, W_dmso_, P_dmso_, N_dmso_ and I_dmso_ are all the same. Combining Equations (4) and (5) resulted in Equation (2). The exact amount of DMSO-*d*_6_ is cancelled and not necessary. DMSO-*d*_6_ function as a bridge to link naproxen and impurity in various NMR solutions.

In Step 2, approximately 0.6 mL of each solution was used for qNMR experiments, and the remaining solutions were mixed in equal proportions for the HPLC experiments. In Step 3, we calculated the RRFs for naproxen impurities using Equation (3) by combining the A_(n,imp)_ provided by the HPLC method.

(4) $$\frac{W_{\mathrm{imp}}\;\times \;P_{\mathrm{imp}}}{W_{\mathrm{dmso}}\;\times \;P_{\mathrm{dmso}}}=\frac{I_{\mathrm{imp}}\;\times \;N_{\mathrm{dmso}}\;\times {\;M}_{\mathrm{imp}}}{I_{\mathrm{dmso}}\;\times \;N_{\mathrm{imp}}\;\times \;M_{\mathrm{dmso}}}$$

(5) $$\frac{W_{n}\;\times \;P_{n}}{W_{\mathrm{dmso}}\;\times \;P_{\mathrm{dmso}}}=\frac{I_{n}\;\times \;N_{\mathrm{dmso}}\;\times \;M_{n}}{I_{\mathrm{dmso}\;}\times \;N_{n}\;\times \;M_{\mathrm{dmso}}}$$

### 2.2. Quantitative Signals and Experimental Parameters Selection

**Figure 3:**
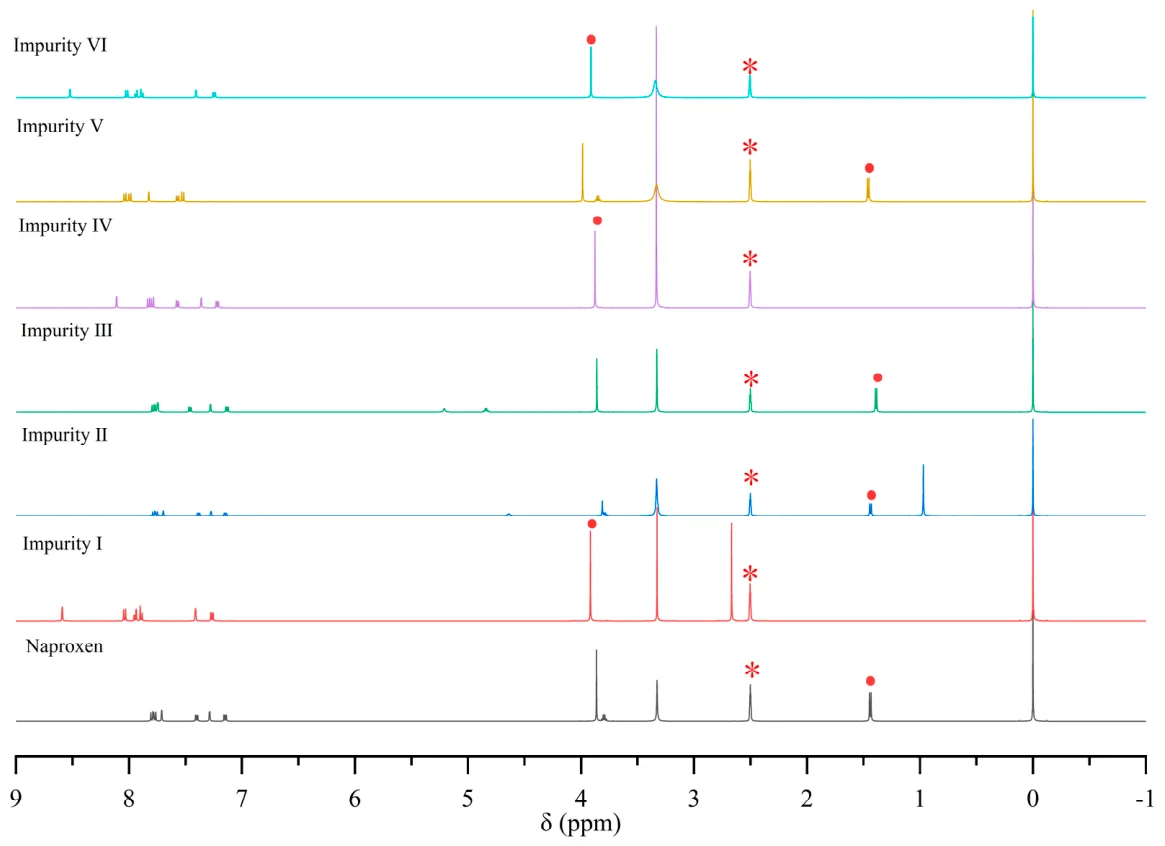
Individual ^1^H NMR spectra of naproxen and impurities I–VI (*: DMSO signal, ●: sample quantitation signal).

In qNMR, the accurate selection of quantitative signals is crucial for reliable data analysis. The δ 2.5 ppm peak of the ^1^H residual signal of DMSO-*d*_6_ is a stable signal with high reproducibility and consistency. It ensures the reliability of qNMR experiment results, eliminating the need to add additional internal standards in qNMR experiments and thereby simplifying the procedure. The naproxen and impurity peaks did not interfere with the DMSO peak at δ 2.5 ppm, ensuring the accuracy of the integration results. Additionally, DMSO exhibited minimal retention on the HPLC columns; therefore, its peak did not interfere with the peaks of the components in the HPLC analysis. In the solutions of naproxen and various impurities, DMSO δ 2.5 ppm (red asterisk) was selected as the internal standard signal peak (Figure 3). Naproxen δ 1.44 ppm (3H), impurity I δ 3.92 ppm(3H), impurity II δ 1.44 ppm (3H), impurity III δ 1.39 ppm (3H), impurity IV δ 3.88 ppm (3H), impurity V δ 1.46 ppm (3H), and impurity VI δ 3.91 ppm (3H) were selected as quantitative peaks (red dots) because they showed good resolution and did not interfere with each other. The signal-to-noise ratio required for quantification was satisfied.

In addition to signal peak selection, the experimental parameters were carefully considered. qNMR experiments require that the relaxation time (D1) is not less than five times the longitudinal relaxation time (T1) [24,25]. The T1 values of the quantitative residual ^1^H signals of deuterium DMSO, naproxen, and impurities I, II, III, IV, V, and VI were determined by the inversion recovery method and were 16.210, 1.284, 1.049, 1.316, 1.677, 1.162, 0.503, and 1.399 s, respectively. To ensure the accuracy of the experimental results, D1 was set to 90 s.

### 2.3. QNMR-HPLC Determines the RRF Using the ^1^H Residual Signal of Deuterated DMSO as an Internal Calibrant

Seven qNMR solutions and one mixed HPLC solution were prepared and tested under the qNMR and HPLC experimental conditions. For each qNMR spectrum, the integral area of the ^1^H residual signal of DMSO-*d*_6_ at δ 2.5 ppm was manually set to 1, and the signal areas of naproxen and impurities I to VI in seven NMR tubes were recorded separately; the results were 0.7669, 0.9313, 0.6276, 0.8902, 0.8591, 0.6290, and 0.8969, respectively. Combined with the signal area corresponding to the HPLC result (injection volume 8 μL), it was calculated according to Equation (3). The RRFs of naproxen impurities I–VI were 0.30, 0.76, 1.14, 1.00, 0.59, and 0.51, respectively. Weighing and purity should not be considered throughout RRF calculations, and there is no need for multiple dilutions, thereby reducing errors in the results.

### 2.4. RRFs from HPLC Standard Curve Method

**Figure 4:**
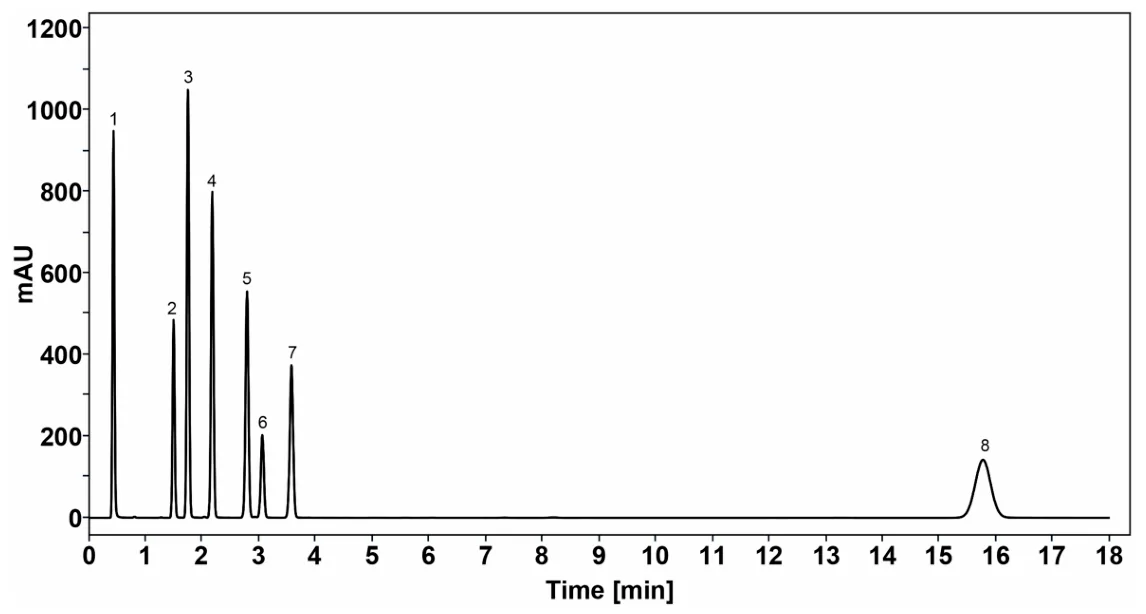
HPLC chromatogram of DMSO-*d*_6_ (1), naproxen (4), impurity I (6), II (5), III (3), IV (8), V (7), and VI (2).

The RRFs of impurities I–VI were evaluated using an HPLC standard curve. A mixed solution of naproxen and impurities of known accurate concentrations was injected and analyzed by HPLC. The chromatograms of naproxen and impurities are shown in Figure 4. The RRFs of the naproxen impurities were 0.30, 0.78, 1.04, 1.05, 0.59, and 0.51, respectively. The HPLC standard curve method results were similar to the qNMR-HPLC using the ^1^H residual signal of DMSO-*d*_6_ as an internal standard. However, this method requires the accurate concentration of the analyte, and when the concentration is not accurate, it directly affects the slope of the linear equation, thus affecting the results of the RRF.

### 2.5. Potential Application of qNMR-HPLC Using the ^1^H Residual Signal of Deuterated DMSO as an Internal Calibrant

Using the ^1^H residual signal of deuterated DMSO as an internal calibrant reduced the purity requirements of the reference material and solved the problem of spectral overlap caused by the determination of multiple impurity RRFs. Accurate weighing of the substance to be measured is not required, which reduces experimental errors caused by weighing. This facilitates determination of the RRFs of multiple impurities quickly, thereby avoiding the complex and time-consuming processes of traditional methods. It is particularly suitable for residual samples on container walls and unstable compounds and offers a fast and environmentally friendly method. For rare or expensive compounds such as novel drugs or costly impurity standards, traditional methods may struggle to accurately generate standard curves because of limited sample quantities. In contrast, using the ^1^H residual signal of deuterated DMSO as an internal calibrant allows analysis even with a limited sample mass, enhancing experimental efficiency and maximizing sample utilization.

This proves the feasibility of using DMSO-*d*_6_ as solvent and its residual protonated DMSO as an internal calibrant. Due to the requirement of qNMR experiments, 90 s D1 and milligram sample amout are utilized in this study. Long experiment time may limit the broader application of this method. Future optimization may be required to further reduce qNMR time and reduce qNMR concertation.

The benchtop NMR instrument has obvious advantages in terms of cost and portability compared with the traditional large-scale NMR instrument. In the future, the combination of benchtop NMR and HPLC technology can also be applied to determine the RRFs of impurities, which has the advantages of rapidity and low cost.

## 3. Materials and Methods

### 3.1. Materials

Naproxen and impurities I–VI were obtained from the National Institutes for Food and Drug Control (Beijing, China); DMSO-*d*_6_ was purchased from Cambridge Isotope Laboratories (Andover, CA, USA); HPLC-grade acetonitrile was obtained from Fisher Scientific International (Waltham, MA, USA); analytical-grade phosphoric acid was obtained from Titan Scientific Co. (Shanghai, China); and analytical-grade potassium dihydrogen phosphate was obtained from Sinopharm Chemical Reagent Co. (Shanghai, China).

### 3.2. ^1^H NMR Measurements

The ^1^H-NMR spectrum was acquired at 298 K using a Bruker 500 MHz nuclear magnetic resonance spectrometer (Bruker, Karlsruhe, Germany). The specific test parameters were set as follows: 16 scans (NS), two dummy scans (DS), 3.27 s acquisition time (AQ), 65 k time domain (TD), 90° excitation pulse, and 90 s relaxation delay (D1). The acquired data were integrated after Fourier transformation and phase and baseline correction using the TopSpin 3.6.5 software (Bruker, Germany).

### 3.3. HPLC Measurements

HPLC analysis was performed on an Agilent 1100 high-performance liquid chromatograph (Santa Clara, CA, USA) and a HALO 90 Å C18 (4.6 × 100 mm, 2.7 μm) column (Wilmington, DE, USA). Isocratic elution was performed according to the Pharmacopoeia of The People’s Republic of China [26]. Mobile phase A was 1.36 g/L solution of potassium dihydrogen phosphate adjusted to pH 2.0 with 85% phosphoric acid, and mobile phase B was acetonitrile (ACN). The ratio of mobile phases A and B was 62:38. Flow rate was set to 2.0 mL/min. The injection volumes were 2, 4, 6, 8, and 10 μL. The column temperature was 50 °C, and the detection wavelength was 230 nm.

### 3.4. Sample Preparation for ^1^H NMR

Approximately 6 mg of naproxen and impurities I, II, III, IV, V, and VI were dissolved in 1 mL of deuterium DMSO to obtain solutions 0, 1, 2, 3, 4, 5, and 6, respectively. Each solution was transferred to 5 mm NMR tubes for qNMR analysis.

### 3.5. Sample Preparation for HPLC

A sample (2 mL) of each solution (0, 1, 2, 3, 4, 5, and 6) was measured in the same 25 mL volumetric flask. The resulting solution was then diluted with HPLC mobile phase to obtain a mixed HPLC solution with a concentration of approximately 0.048 mg/mL for naproxen and impurities I, II, III, IV, V, and VI.

## 4. Conclusions

In this study, a qNMR-HPLC technology using the ^1^H residual signal of deuterated DMSO as an internal calibrant was achieved. The RRFs of naproxen impurities I–VI were calculated, and the results closely aligned with those obtained using the HPLC standard curve method. Methodological validation indicated that the established method exhibited good linearity for naproxen concentration ranging from 1 to 12 mg/mL. This method had good precision for repeated injections, and the solution was stable for 24 h. This study addresses the challenge of overlapping mixed spectra when determining multiple impurity RRFs through qNMR-HPLC, which requires only the separation and synthesis of the target impurity components. This significantly reduces the cost associated with standard substance preparation and shortens the preparation time. It is also particularly suitable for analyzing multiple pharmaceutical impurities and scarce samples, positioning it to play a more pivotal role in future studies and applications. However, when the ^1^H residual signal of DMSO at δ 2.5 ppm interferes with the signal peak of the substance to be measured, it may lead to the inaccurate integration of results, which can affect the reliability of qNMR experiments. In this case, DMSO-*d*_6_ may not be suitable as an internal calibrant, and other internal calibrants should be used. The method relies on the ability to isolate and synthesize target impurity substances. If these impurities cannot be isolated or synthesized, the approach is not applicable. Alternatively, quantum mechanical calculations or the introduction of large artificial intelligence models might be necessary to address this challenge.

## Data Availability

The data presented in this study are available on request from the corresponding author.

## Abbreviations

The following abbreviations are used in this manuscript:

qNMR-HPLC	Quantitative nuclear magnetic resonance coupled with HPLC
RRFs	relative response factors
MS	mass spectrometry
API	active pharmaceutical ingredient
